# The Reliability and Quality of Short Videos as a Source of Dietary Guidance for Inflammatory Bowel Disease: Cross-sectional Study

**DOI:** 10.2196/41518

**Published:** 2023-02-09

**Authors:** Zixuan He, Zhijie Wang, Yihang Song, Yilong Liu, Le Kang, Xue Fang, Tongchang Wang, Xuanming Fan, Zhaoshen Li, Shuling Wang, Yu Bai

**Affiliations:** 1 Department of Gastroenterology, Changhai Hospital Naval Medical University Shanghai China; 2 Changhai Clinical Research Unit Shanghai China; 3 College of Basic Medicine Naval Medical University Shanghai China

**Keywords:** inflammatory bowel disease, diet, information quality, social media, gastroenterology, nutrition, videos, health communication

## Abstract

**Background:**

Dietary management is considered a potential adjunctive treatment for inflammatory bowel disease (IBD). Short-video sharing platforms have enabled patients to obtain dietary advice more conveniently. However, accessing useful resources while avoiding misinformation is not an easy task for most patients.

**Objective:**

This study aimed to evaluate the quality of the information in IBD diet–related videos on Chinese short-video sharing platforms.

**Methods:**

We collected and extracted information from a total of 125 video samples related to the IBD diet on the 3 Chinese short-video sharing platforms with the most users: TikTok, Bilibili, and Kwai. Two independent physicians evaluated each video in terms of content comprehensiveness, quality (rated by Global Quality Score), and reliability (rated by a modified DISCERN tool). Finally, comparative analyses of the videos from different sources were conducted.

**Results:**

The videos were classified into 6 groups based on the identity of the uploaders, which included 3 kinds of medical professionals (ie, gastroenterologists, nongastroenterologists, and clinical nutritionists) and 3 types of non–medical professionals (ie, nonprofit organizations, individual science communicators, and IBD patients). The overall quality of the videos was poor. Further group comparisons demonstrated that videos from medical professionals were more instructive in terms of content comprehensiveness, quality, and reliability than those from non–medical professionals. Moreover, IBD diet–related recommendations from clinical nutritionists and gastroenterologists were of better quality than those from nongastroenterologists, while recommendations from nonprofit organizations did not seem to be superior to other groups of uploaders.

**Conclusions:**

The overall quality of the information in IBD diet-related videos is unsatisfactory and varies significantly depending on the source. Videos from medical professionals, especially clinical nutritionists and gastroenterologists, may provide dietary guidance with higher quality for IBD patients.

## Introduction

Inflammatory bowel disease (IBD) includes two clinical phenotypes, ulcerative colitis (UC) and Crohn disease (CD), both of which are characterized by chronic and relapsing intestinal inflammation [1]. Even though current studies indicate the involvement of immune malfunction, gut microbiota, and genetic variability, the pathogenesis remains largely unclear [2,3]. Moreover, the treatment of IBD is complex, and drug therapy alone may not suffice to provide long-term remission in all patients. Dietary management is considered a potential adjunctive treatment in IBD. A study found that more than 68% of IBD patients used dietary restrictions to control symptoms and avoid progression [4]. However, given the inherent clinical features of IBD, malnutrition in IBD patients appears to be common and complex, with serious adverse consequences [5]. Excessive or irrational dietary restrictive behaviors have significant impact on the social life of IBD patients and even contribute negatively to disease control [6]. A common complaint among IBD patients is the lack of guidance regarding what and how to eat [7,8]. The internet has enabled patients to obtain answers more conveniently. Patients with IBD were active on social media for many years before physicians realized the value and influence of social media in IBD education and care. As a result, patients are more likely to seek dietary advice from social media than medical professionals, a trend that was more pronounced during the COVID-19 pandemic [9].

Previous studies have shown that IBD patients exposed to social media are more likely to engage with IBD-related information [10]. In recent years, social media with visual content, such as YouTube and TikTok, have gradually emerged on the internet. These media seem to have an irreplaceable advantage in health information communication. Graphical video information is more easily absorbed and remembered than textual information, helping patients make judgments about their health status and tailor their diet accordingly [11,12]. However, assessing the quality of dietary recommendations on the internet is not an easy task for most IBD patients [13,14]. The quality of online information about IBD varies widely. For a significant portion of the patient population, online information is too complex to comprehend, and good-quality information may be out of reach for most IBD patients [15].

A recent study of internet use by patients with IBD revealed that over half of patients considered the internet to be the most common source of information, and the majority of patients rated internet information as “trustworthy” or “very trustworthy” [16]. However, we must recognize that many medical science videos come from lay users without medical professional training, which leads to mixed information and inaccurate or biased information that may mislead patients or even have a negative effect on their health [17,18]. Therefore, health care practitioners should assess the quality of online information and inform their patients. Short-video sharing platforms, such as TikTok, Bilibili, and Kwai, provide abundant information resources and have attracted more than 500 million users in China with their convenience, interactivity, and diversity. On these platforms, patients can access a large number of health videos, including IBD-related diet-guidance videos, without registration or payment simply by typing in keywords for their topic of interest in the search box. However, to the best of our knowledge, the quality of dietary-related information for IBD patients in video-based social media has not been adequately evaluated. This study aims to fill this information gap by assessing the information quality of IBD diet-related videos on TikTok, Bilibili, and Kwai.

## Methods

### Search Strategy and Data Extraction

**Figure 1:**
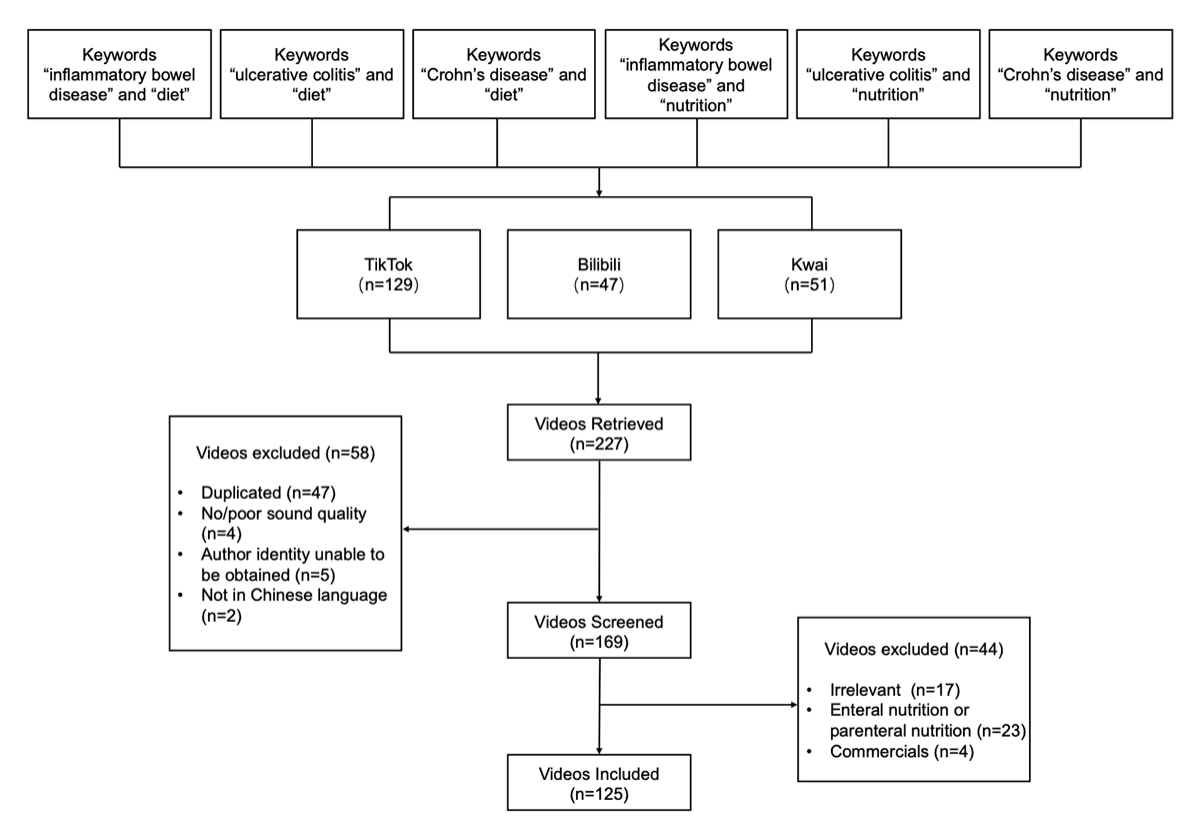
Search strategy and video screening procedure.

All collected videos were sourced from TikTok, Bilibili, or Kwai, 3 of the most popular Chinese short-video sharing platforms. The search keywords were 炎症性肠病 (“inflammatory bowel disease”), 溃疡性结肠炎 (“ulcerative colitis”), or 克罗恩病 (“Crohn disease”) combined with “饮食”(“diet”), or “营养”(“nutrition”). The entire search process was conducted and completed between May 3 and May 5, 2022. We included only Chinese-language videos that primarily focused on an IBD (CD or UC) diet. Videos were excluded if they were duplicates, had no sound or poor sound quality, were for commercial purposes, were irrelevant to the topic, if the author identity could not be obtained, or if they were not in Chinese. Videos with multiple parts were counted as a single video. In addition, videos related to enteral or parenteral nutrition for IBD were also excluded (Figure 1). Basic information on the included videos was extracted, including the name and identity of the uploader, the length of the video, and the number of likes it received. All extracted data were recorded in Excel (Microsoft Corp).

### Evaluating Methodologies

The content, reliability, and quality of the videos were evaluated by scoring. Based on the recommendations from the International Organization for the Study of Inflammatory Bowel Disease (IOIBD) and the best available evidence to date [19-21], dietary guidance for IBD patients was summarized according to the following 6 aspects: fruits/vegetables, carbohydrates, meats, fats, alcohol, and food additives. The detailed recommendations are shown in Table 1. The video content score was defined as the total number of accurate recommendations for each of the above aspects, with the highest possible score being 6. The DISCERN tool has been widely validated and applied for evaluating health-related content on video sharing platforms (ie, YouTube, Facebook, and TikTok) [22-24]. We therefore used a modified DISCERN questionnaire to assess the content’s reliability based on 5 aspects: clarity, relevancy, traceability, robustness, and impartiality (Multimedia Appendix 1). The Global Quality Score (GQS), a commonly used 5-point Likert scale ranging from 1 (poor quality) to 5 (excellent quality) for the evaluation of internet videos [24-26], was applied to assess the overall quality of the videos in this study (Multimedia Appendix 2).

**Table 1:** Detailed content evaluation of diet recommendations for patients with inflammatory bowel disease.

Aspects of diet	Recommendations
Fruits and vegetables	Adequate daily intake of fruits and vegetables.
Carbohydrates	Adequate daily intake of all carbohydrates, including gluten-containing foods.
Meats	Moderate intake of red meat, chicken, and fish, with less intake of processed meats.
Fats	Consumption of less saturated fat/myristic acid, avoidance of trans fats, and consumption of more wild fish rich in omega-3 fatty acid.
Alcohol	Low intake of alcoholic beverages.
Food additives	Limited intake of foods containing food additives, including maltodextrin, artificial sweeteners, emulsifiers, and thickeners.

### Evaluation Procedure

To minimize bias introduced by personal recommendation algorithms, new accounts were registered and logged for each video platform. The evaluation tasks were accomplished by 2 qualified physicians (ZH and ZW) working in the division of digestive disease in a tertiary teaching hospital. All videos were browsed without downloading, reposting, liking, or commenting. Before starting to score the videos, the 2 raters first reviewed dietary guidance from the IOIBD [19] and official DISCERN and GQS scoring instructions; they then discussed how the tool could be operationalized for evaluating video-based content and made necessary adjustments. Each video was evaluated by the 2 raters, followed by discussion and resolution of any inconsistencies. Cohen κ coefficients were calculated to determine the interrater reliability. The interrater reliability for each evaluation item was greater than 0.8, indicating good interrater reliability. For those scores on which agreement could not be reached, the final decision was made by a senior author (YB or ZL). Comparisons between groups of 2 were performed using nonparametric Mann-Whitney tests, while comparisons among groups of 3 were made with the Kruskal-Wallis H test. R software (version 3.6.3; R Foundation for Statistical Computing) was used for statistical analysis and data visualization.

## Results

### Video Characteristics

After the inclusion and exclusion criteria were applied, a total of 125 videos were included for further data extraction and analysis (Figure 1). We classified the 125 postscreening videos into 2 groups based on the identity of the uploaders (medical professionals vs non–medical professionals). Among videos uploaded by medical professionals, 3 types of video creators were identified with different medical specialties: gastroenterologists, nongastroenterologists, and clinical nutritionists. Among videos from non–medical professionals, we also identified 3 types of video creators: nonprofit organizations, individual science communicators, and patients with IBD. As shown in Table 2, 72 of the 125 videos were shared by medical professionals (58%), whereas 53 were shared by non–medical professionals (42%). Among medical professionals, nongastroenterologists contributed the most videos (n=42, 34%), followed by gastroenterologists (n=26, 21%) and clinical nutritionists (n=4, 3%), while among the creators of videos from non–medical professionals, the most videos were uploaded by individual science communicators (n=22, 18%), followed by non-profit organizations (n=18, 14%) and IBD patients (n=13, 10%). Across all included videos, the median duration of the videos was 69 (IQR 43-116) seconds and the median number of likes received was 47 (IQR 8-118). Interestingly, we found that videos uploaded by medical professionals had a shorter duration (median 60, IQR 27-93 seconds) but received more likes (median 71, IQR 13-287) than videos from non–medical professionals (median duration 96, IQR 59-141 seconds; median likes received 10, IQR 4-87).

**Table 2:** Characteristics of the videos across sources.

Source (Description)			Video duration in seconds (mean total 69, IQR 43-116), median (IQR)		Number of likes (median total 47, IQR 8/118), median (IQR)		Videos, n (%)
**Medical professionals**							
	Gastroenterologists (doctors who specialize in gastroenterology)	71 (47-93)		88 (9-289)		26 (21)	
	Nongastroenterologists (doctors who specialize in medical fields other than gastroenterology)	53 (34-88)		71 (30-287)		42 (34)	
	Clinical nutritionists (professionals who provide nutrition or diet advice for patients)	118 (52-220)		78 (25-107)		4 (3)	
	Overall	60 (27-93)		71 (13-287)		72 (58)	
**Non–medical professionals**							
	Nonprofit organizations (public accounts operated by organizations)	97 (45-109)		10 (7-87)		18 (14)	
	Individual science communicators (general users who participate in general scientific communications)	67 (38-121)		6 (2-50)		22 (18)	
	Patients (patients with inflammatory bowel disease)	125 (98-278)		92 (5-216)		13 (10)	
	Overall	96 (59-141)		10 (4-87)		53 (42)	

### Video Content

**Figure 2:**
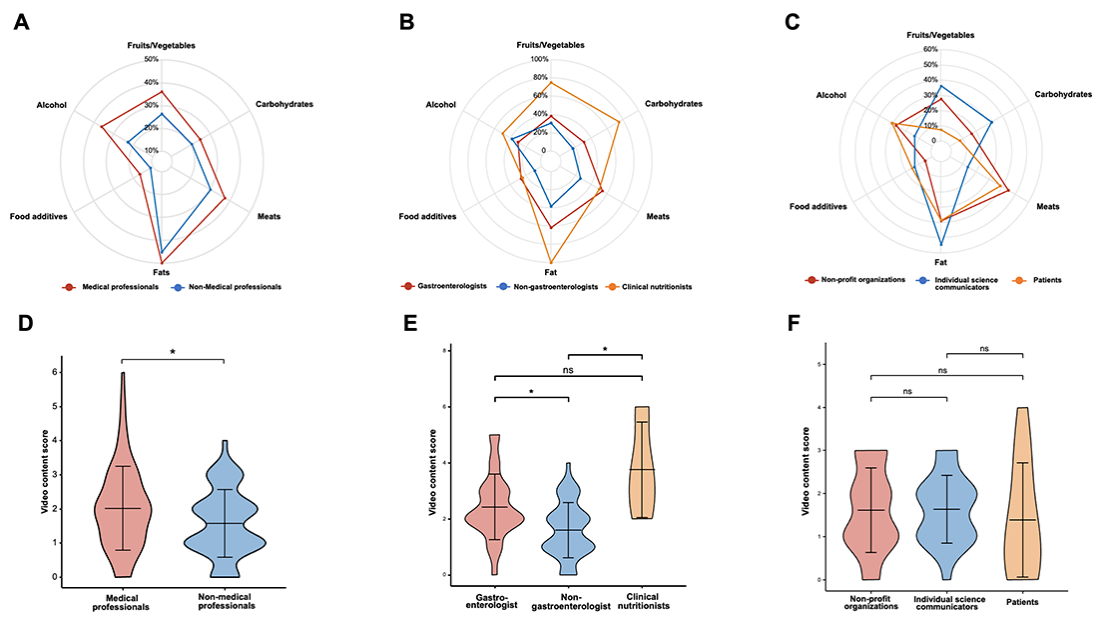
Comparison of content comprehensiveness between sources. (A-C) Radar charts showing the percentage of each inflammatory bowel disease diet–related recommendation among videos from different sources. (D-E) Violin plots showing the total content scores among videos from different sources. *P<.05; ns: nonsignificant.

We evaluated the content comprehensiveness of each video; the results showed that very few videos could provide comprehensive guidance on diet for patients with IBD. Almost half of the 125 videos (n=60, 48%) offered recommendations on fat consumption for patients with IBD based on guidance from the IOIBD, followed by recommendations on meat (n=43, 34%), fruits and vegetables (n=40, 32%), alcohol (n=38, 30%), and carbohydrates (n=29, 23%), while for food additives, only 14% (n=18) of the videos addressed the topic and gave the appropriate recommendations. Next, we compared content comprehensiveness across the sources of videos. As is shown in Figure 2A and Figure 2D, videos from medical professionals had a higher coverage proportion for all 6 aspects of content, as well as a higher overall content score, than videos from non–medical professionals. Furthermore, we evaluated the video content of each subgroup of medical professionals and non–medical professionals. As illustrated in Figure 2B and Figure 2E, clinical nutritionists appeared to perform the best among medical professionals in terms of comprehensiveness of video content, providing more diet advice on fruits and vegetables, carbohydrates, fats, and alcohol for patients with IBD. For the overall video content scores, clinical nutritionists and gastroenterologists rated higher than nongastroenterologists. In videos from non–medical professional sources, individual science communicators provided more guidance on fruits and vegetables, carbohydrates, and fat intake, while videos from nonprofit organizations and patients with IBD placed more emphasis on alcohol and meat consumption. In terms of video content scores, the 3 groups of video sources did not show significant differences (Figure 2C and Figure 2F).

### Information Quality and Reliability

**Figure 3:**
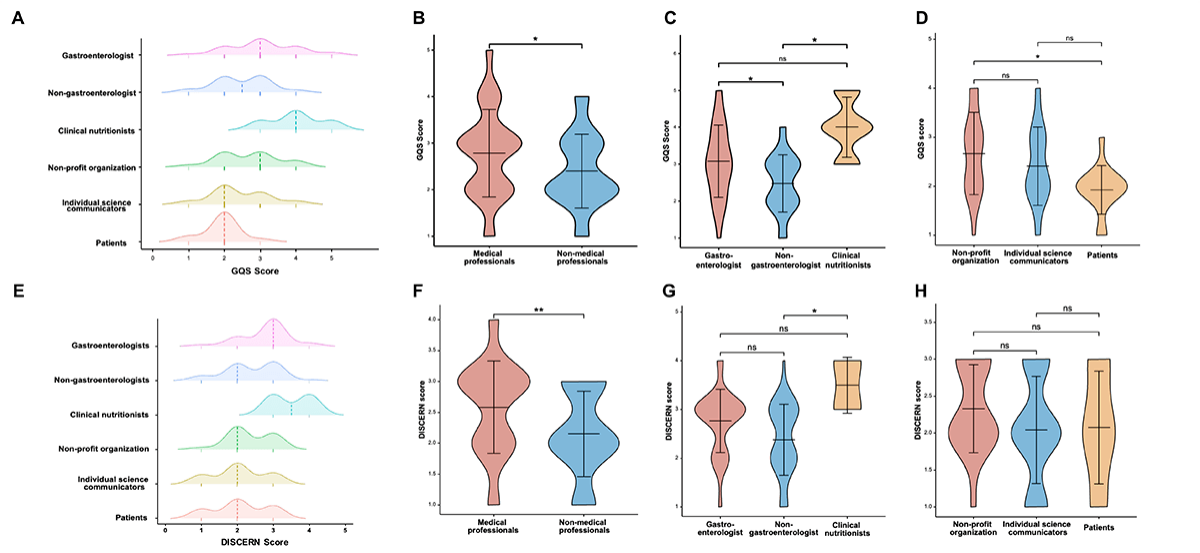
Comparisons of Global Quality Score and DISCERN score among different sources. (A) Ridge plot showing the overall distribution of Global Quality Score among different sources. (B-D) Violin plots showing the Global Quality Score among videos from different sources. (E) Ridge plot showing the overall distribution of DISCERN scores among different sources. (F-H) Violin plots showing the DISCERN scores among videos from different sources. GQS: Global Quality Score. *P<.05; **P<.01; ns: non-significant.

We first assessed the general quality of each video using the GQS scale; as shown in Table 3, the mean GQS value for all videos was 2.61 (SD 0.9), with a median score of 3 (IQR 2-3). In addition, the GQS values of videos from medical professionals were significantly higher than those from non–medical professionals. Further subgroup analysis revealed that among the videos from medical-professional sources, the quality of videos provided by clinical nutritionists and gastroenterologists was significantly higher than nongastroenterologists (*P*=.01 and .04, respectively). Moreover, in videos from non–medical professionals, the quality of videos from IBD patients was relatively lower than nonprofit organizations (*P*=.02; Figures 3A-D). Regarding the reliability of the videos, the mean score for all videos was 2.15 (SD 0.69), with a median score of 2 (IQR 2-3) (Table 3). Consistent with the results for the GQS, the reliability scores of videos from medical professionals were significantly higher in comparison to videos from non–medical professionals. Further analysis revealed that among the videos from medical professionals, those provided by clinical nutritionists were of significantly higher quality (*P*=.01 compared to non-gastroenterologists). (Figures 3E-H). The reliability scores of videos from the 3 non–medical-professional sources did not differ significantly.

**Table 3:** Global Quality Score and DISCERN scores for inflammatory bowel disease diet–related videos by source.

Source		GQS^a^ scores (total 3, IQR 2-3), median (IQR)	GQS scores (total 2.61, SD 0.90), mean (SD)	DISCERN scores (total 2, IQR 2-3), median (IQR)	DISCERN scores (total 2.40, SD 0.75), mean (SD)
**Medical professionals**					
	Gastroenterologists	3 (2-4)	3.08 (0.98)	3 (2-3)	2.77 (0.65)
	Nongastroenterologists	3 (2-3)	2.48 (0.77)	2 (2-3)	2.38 (0.73)
	Clinical nutritionists	4 (3-5)	4.00 (0.82)	4 (3-4)	3.50 (0.58)
	Overall	3 (2-3)	2.78 (0.94)	3 (2-3)	2.58 (0.74)
**Non–medical professionals**					
	Nonprofit organizations	3 (2-3)	2.67 (0.84)	2 (2-3)	2.33 (0.59)
	Individual science communicators	2 (2-3)	2.41 (0.80)	2 (2-3)	2.05 (0.72)
	Patients	2 (2-2)	1.92 (0.49)	2 (2-3)	2.08 (0.76)
	Overall	2 (2-3)	2.40 (0.79)	2 (2-3)	2.15 (0.69)

^a^GQS: Global Quality Score.

## Discussion

### Principal Findings

In this study, we reviewed the 3 most popular Chinese short-video sharing platforms: TikTok, Bilibili, and Kwai. We evaluated the content, quality, and reliability of all videos on the topic of IBD diet. In general, the overall quality of these videos was not satisfactory, which is probably due to the fact that health-related information on these platforms is not regulated or monitored before being posted. In addition, the quality of the videos varied significantly depending on the source. Our results showed that very few videos were comprehensive enough to cover all components of daily diet for patients with IBD and provide appropriate and trustworthy recommendations. This was especially the case for food additives and carbohydrate intake, as less than 30% of the videos addressed these topics and provided correct dietary advice. Meanwhile, during our review of the video content, we found that some videos provided inaccurate dietary recommendations; for example, 5 videos mentioned that patients with IBD at any stage should completely avoid fiber-rich vegetables and whole-grain carbohydrates, while 7 videos stated that patients with IBD should avoid dairy intake. These recommendations are contrary to guidance from the IOIBD and are not backed by evidence. Nutritional supplementation, including with vitamins and micronutrients such as calcium, iron, and zinc, for patients with IBD is also an important issue, but it is noteworthy that according to our search results, there were few videos addressing and providing recommendations on this issue, so we did not analyze or discuss this content further. Given the increasing availability and promotion of nutritional supplements on the market today, the discussion and interpretation of this issue deserves more attention.

Previous studies have indicated that the overall quality of health education videos varied according to the identity of the author [23,27]. Our results suggest that videos from medical professionals, especially clinical nutritionists and gastroenterologists, had comparatively higher guidance value than those from non–medical professionals in terms of content comprehensiveness, quality, and reliability of content. This may be attributed to the fact that medical professionals are well-informed about relevant IBD dietary guidelines, the current consensus, and the literature, and they are more sensitive to updated knowledge, whereas non–medical professionals, such as IBD patients, rely more on their own experience and personal insights, which may be biased to some extent [28,29]. Nonprofit organizations, such as educational institutions and medical institutions, are generally considered to be reliable sources of health information [23]. However, in our study, IBD diet–related recommendations from nonprofit organizations did not appear to be more instructive than those from other groups of uploaders, and we also noticed that a large amount of professional jargon was used in videos from nonprofit organizations, which might make them difficult for a large proportion of patients to understand.

### High-Quality Health Education Can Promote Self-Management Abilities in Patients with IBD

Improved knowledge of IBD, its management, and the principles of its treatment may lead to better disease outcomes and a decrease in the impact of this disease on daily life [30-32]. According to the World Health Organization, “health education has the objective of helping patients acquire or maintain skills that they need to best manage their life with a chronic disease.” High-quality health education helps patients understand their illness, work together, and accept responsibility for their own care, so that they become active participants in their own treatment. For IBD in particular, high-quality health education can help not only improve patients’ awareness of the disease and their self-management ability, but also effectively reduce the risk of recurrence and related complications [33,34]. A number of recent studies have also demonstrated that appropriate education is an effective method for reducing inappropriate steroid use and psychological distress, improving self-management skills in IBD patients [33,35,36].

### Dietary Management Plays a Vital Role in the Treatment of Patients with IBD

Dietary therapy has long been accepted as a classic treatment modality by a majority of patients, especially those with digestive diseases [37]. Diet for patients with IBD has been an expanding area of research in recent years [38,39]. Many components of daily diet have been found to contribute to the deterioration and recurrence of IBD. In addition, an increasing amount of evidence suggests that unhealthy dietary habits, such as Western dietary patterns and excessive intake of ultraprocessed foods, are closely associated with a worse prognosis for IBD [40-42]. Despite the fact that some kinds of food are considered to be triggers of intestinal symptoms in patients with IBD, undifferentiated exclusion of certain nutrients may result in severe nutritional deficiencies, which in turn may lead to malnutrition and increased risk of hospitalization [43]. In particular, milk and dairy products, which represent the main sources of calcium and vitamin D, are the foods most frequently avoided by patients, especially during disease flare-ups [44,45].

In 2020, the nutrition cluster of the IOIBD developed an expert consensus on IBD diet based on the best current evidence, which included specific dietary components and food groups in the daily diet and provided detailed recommendations for the diet of IBD patients. Nevertheless, despite the availability of these expert authority opinions and guidelines, it is difficult for most patients and family members without a medical background to learn about these recommendations through appropriate and convenient sources. The advent of the internet has removed obstacles to health information communication; this is especially the case for certain websites and mobile apps, including TikTok, that use a video format. There is strong evidence that COVID-19–related videos on TikTok were viewed at least 93.1 billion times during the pandemic by July 2020 [46]. However, easy access to health information is always accompanied by the dissemination of a large amount of low-quality, scientifically unsupported, and even erroneous health information [47]. Thus, it is necessary for health practitioners to screen videos on the Web containing health information for content and quality to provide patients with search guidance [24].

### Practical Significance

It is quite common for patients to use the internet as a source of information for disease self-management, especially patients with chronic illnesses such as IBD and diabetes mellitus. Videos are generally considered to present complex health information in a more comprehensible and impressive way when compared with text. Thus, social media with visual content is gradually becoming an important information source for patients. On the other hand, these platforms are also powerful ways for health care practitioners to reach and educate their patients. In fact, the positive role of video education is supported by a growing body of evidence. Compared with a written pamphlet, online video-based education was shown to markedly improve disease knowledge and clinical outcomes among patients with atopic dermatitis in a randomized controlled trial [48]. In another randomized controlled trial recently published by Molavynejad et al [49], weight, glycemic parameters, and lipid profiles significantly decreased in a video-education group compared to a control group (education was carried out by staff nurses via pamphlets). Moreover, digital health may provide advantages in value-based care and population health management for patients with IBD. Several randomized controlled trials have demonstrated that digital health interventions (ie, educational videos, mobile apps, and telemedicine) may reduce overall health care resource use compared to standard care, primarily by decreasing outpatient visits for patients with IBD [50-53]. Moreover, among studies assessing the impact of digital health technologies on health care costs, 3 studies showed that web-based interventions or telephone consultations resulted in significant cost savings compared to traditional face-to-face encounters [54-56]. Overall, the evidence suggests that there is huge potential for digital health in the management of chronic diseases such as IBD; this calls for more high-quality, well-designed studies with a broader coverage of the patient population in the future, as well as policies that encourage patient engagement in digital health and improve the efficiency of care.

### Limitations and Future Directions

Internet-based health promotion has become a topic of increasing attention, and a guideline on publishing and disseminating health-science knowledge through various media was recently issued by the Chinese government. However, there is no formal guideline focused on health-promoting videos anywhere in the world, as far as we know. Considering the increased popularity of video-sharing platforms, the essential criteria for content on these platforms should be discussed. In any case, health practitioners and video-sharing platform operators should be the first to act to change this situation. The platforms should be encouraged to set up health sections that are separate from other videos. Only videos audited by professionals or made by verified medical professionals should be allowed to be uploaded in this section. Alternatively, although videos have overcome educational barriers to a certain extent by presenting complex information in an easier-to-access way, it is still difficult for many audiences to understand professional vocabulary due to the complexity of medicine. Thus, medical professionals should be taught to make their videos more comprehensible, while non–medical professionals should be requested to present evidence-based information as much as possible. Excellent health-promoting videos must balance scientific soundness, popularity, and ease of understanding. Finally, given that good and bad videos are currently intermingled, it is necessary for health practitioners to screen videos containing health information for content and quality to provide patients with guidance.

There are still limitations to be considered in this study. First, we only included videos uploaded on Chinese video-sharing platforms, so the findings may not be generalizable to platforms in other languages (eg, YouTube). Second, there were uncertainties in this study due to a small sample size of videos by clinical nutritionists. In general, more cross-language comparative studies with larger sample sizes will be necessary in the future to confirm our findings.

### Conclusion

In this study, 125 IBD diet-related videos from 3 short-video sharing platforms (TikTok, Bilibili, and Kwai) were evaluated for their information quality. The results demonstrated that the quality of these videos was unsatisfactory and varied widely depending on the type of source. Overall, videos from medical professionals were more instructive in terms of comprehensiveness of content, quality, and reliability than those from non–medical professionals. Moreover, IBD diet recommendations from clinical nutritionists and gastroenterologists were of better quality than those from nongastroenterologists, while recommendations from nonprofit organizations did not seem to be superior to other groups of uploaders. Overall, given the growing popularity of video sharing platforms, discussion of essential criteria should be put on the agenda.

## Appendix

Multimedia Appendix 1 Modified DISCERN quality criteria for assessing the reliability of videos; 1 point for answering yes, 0 points for answering no.

Multimedia Appendix 2 Global Quality Score (GQS) scoring; ranges from 1 to 5.

## Abbreviations

CD: Crohn disease
GQS: Global Quality Score
IBD: inflammatory bowel disease
IOIBD: International Organization for the Study of Inflammatory Bowel Disease
UC: ulcerative colitis

## References

[1] Kaplan GG, Windsor JW (2021). The four epidemiological stages in the global evolution of inflammatory bowel disease. Nat Rev Gastroenterol Hepatol.

[2] Graham DB, Xavier RJ (2020). Pathway paradigms revealed from the genetics of inflammatory bowel disease. Nature.

[3] Ananthakrishnan AN, Bernstein CN, Iliopoulos D, Macpherson A, Neurath MF, Ali RAR, Vavricka SR, Fiocchi C (2018). Environmental triggers in IBD: a review of progress and evidence. Nat Rev Gastroenterol Hepatol.

[4] Limdi JK, Aggarwal D, McLaughlin JT (2016). Dietary practices and beliefs in patients with inflammatory bowel disease. Inflamm Bowel Dis.

[5] Roncoroni L, Gori R, Elli L, Tontini GE, Doneda L, Norsa L, Cuomo M, Lombardo V, Scricciolo A, Caprioli F, Costantino A, Scaramella L, Vecchi M (2022). Nutrition in patients with inflammatory bowel diseases: a narrative review. Nutrients.

[6] Zallot C, Quilliot D, Chevaux J, Peyrin-Biroulet C, Guéant-Rodriguez Rosa Maria, Freling E, Collet-Fenetrier B, Williet N, Ziegler O, Bigard M, Guéant Jean-Louis, Peyrin-Biroulet L (2013). Dietary beliefs and behavior among inflammatory bowel disease patients. Inflamm Bowel Dis.

[7] Czuber-Dochan W, Morgan M, Hughes LD, Lomer MCE, Lindsay JO, Whelan K (2020). Perceptions and psychosocial impact of food, nutrition, eating and drinking in people with inflammatory bowel disease: a qualitative investigation of food-related quality of life. J Hum Nutr Diet.

[8] Peters V, Alizadeh BZ, de Vries JH, Dijkstra G, Campmans-Kuijpers MJ (2019). Nutritional Assessment in Inflammatory Bowel Disease (IBD)-Development of the Groningen IBD Nutritional Questionnaires (GINQ). Nutrients.

[9] Ahmed W, Taft TH, Charabaty A (2021). Social media in inflammatory bowel disease: the patient and physician perspective. Curr Opin Gastroenterol.

[10] Reich J, Guo L, Groshek J, Weinberg J, Chen W, Martin C, Long MD, Farraye FA (2019). Social media use and preferences in patients with inflammatory bowel disease. Inflamm Bowel Dis.

[11] Feng B, Malloch YZ, Kravitz RL, Verba S, Iosif A, Slavik G, Henry SG (2021). Assessing the effectiveness of a narrative-based patient education video for promoting opioid tapering. Patient Educ Couns.

[12] Rus HM, Cameron LD (2016). Health communication in social media: message features predicting user engagement on diabetes-related Facebook pages. Ann Behav Med.

[13] Song S, Zhang Y, Yu B (2021). Interventions to support consumer evaluation of online health information credibility: A scoping review. Int J Med Inform.

[14] Sun Y, Zhang Y, Gwizdka J, Trace CB (2019). Consumer evaluation of the quality of online health information: systematic literature review of relevant criteria and indicators. J Med Internet Res.

[15] van der Marel S, Duijvestein M, Hardwick JC, van den Brink GR, Veenendaal R, Hommes DW, Fidder HH (2009). Quality of web-based information on inflammatory bowel diseases. Inflamm Bowel Dis.

[16] Włodarczyk M, Włodarczyk J, Zalewska K, Olczyk M, Maryńczak K, Gajewski P, Małek Z, Cetnar-Sokołowska Z, Sobolewska-Włodarczyk A, Dziki A, Dziki (2019). Preferences of patients with inflammatory bowel disease for receiving specialized health services using technology: the role of Internet and other sources of medical information. Pol Przegl Chir.

[17] Scanfeld D, Scanfeld V, Larson EL (2010). Dissemination of health information through social networks: twitter and antibiotics. Am J Infect Control.

[18] Fortinsky KJ, Fournier MR, Benchimol EI (2012). Internet and electronic resources for inflammatory bowel disease: a primer for providers and patients. Inflamm Bowel Dis.

[19] Levine A, Rhodes JM, Lindsay JO, Abreu MT, Kamm MA, Gibson PR, Gasche C, Silverberg MS, Mahadevan U, Boneh RS, Wine E, Damas OM, Syme G, Trakman GL, Yao CK, Stockhamer S, Hammami MB, Garces LC, Rogler G, Koutroubakis IE, Ananthakrishnan AN, McKeever L, Lewis JD (2020). Dietary guidance from the International Organization for the Study of Inflammatory Bowel Diseases. Clin Gastroenterol Hepatol.

[20] Day AS, Wood JA, Halmos EP, Bryant RV (2021). Practical guidance for dietary management of patients with inflammatory bowel disease during the SARS-CoV2 pandemic. J Acad Nutr Diet.

[21] Halmos EP, Gibson PR (2015). Dietary management of IBD--insights and advice. Nat Rev Gastroenterol Hepatol.

[22] Singh AG, Singh S, Singh PP (2012). YouTube for information on rheumatoid arthritis--a wakeup call?. J Rheumatol.

[23] Song S, Xue X, Zhao YC, Li J, Zhu Q, Zhao M (2021). Short-video apps as a health information source for chronic obstructive pulmonary disease: information quality assessment of TikTok videos. J Med Internet Res.

[24] Langille M, Bernard A, Rodgers C, Hughes S, Leddin D, van Zanten SV (2010). Systematic review of the quality of patient information on the internet regarding inflammatory bowel disease treatments. Clin Gastroenterol Hepatol.

[25] Mueller SM, Jungo P, Cajacob L, Schwegler S, Itin P, Brandt O (2019). The absence of evidence is evidence of non-sense: cross-sectional study on the quality of psoriasis-related videos on Youtube and their reception by health seekers. J Med Internet Res.

[26] Mukewar S, Mani P, Wu X, Lopez R, Shen B (2013). YouTube and inflammatory bowel disease. J Crohns Colitis.

[27] Kong W, Song S, Zhao YC, Zhu Q, Sha L (2021). TikTok as a health information source: assessment of the quality of information in diabetes-related videos. J Med Internet Res.

[28] Berland GK, Elliott MN, Morales LS, Algazy JI, Kravitz RL, Broder MS, Kanouse DE, Muñoz J A, Puyol JA, Lara M, Watkins KE, Yang H, McGlynn EA (2001). Health information on the Internet: accessibility, quality, and readability in English and Spanish. JAMA.

[29] Walji M, Sagaram S, Sagaram D, Meric-Bernstam F, Johnson C, Mirza NQ, Bernstam EV (2004). Efficacy of quality criteria to identify potentially harmful information: a cross-sectional survey of complementary and alternative medicine web sites. J Med Internet Res.

[30] Robinson A, Thompson DG, Wilkin D, Roberts C, Northwest Gastrointestinal Research Group (2001). Guided self-management and patient-directed follow-up of ulcerative colitis: a randomised trial. Lancet.

[31] Tran L, Mulligan K (2019). A systematic review of self-management interventions for children and adolescents with inflammatory bowel disease. Inflamm Bowel Dis.

[32] Waters BM, Jensen L, Fedorak RN (2005). Effects of formal education for patients with inflammatory bowel disease: a randomized controlled trial. Can J Gastroenterol.

[33] Park Y, Choi CH, Kim HS, Moon HS, Kim DH, Kim JJ, Teng D, Park DI (2022). Physician education can minimize inappropriate steroid use in patients with inflammatory bowel disease: the ACTION study. Intest Res.

[34] McDermott E, Healy G, Mullen G, Keegan D, Byrne K, Guerandel A, Forry M, Moloney J, Doherty G, Cullen G, Malone K, Mulcahy H (2018). Patient education in inflammatory bowel disease: a patient-centred, mixed methodology study. J Crohns Colitis.

[35] Zhang Y, Pi B, Xu X, Li Y, Chen X, Yang N (2020). Influence Of narrative medicine-based health education combined with an online patient mutual assistance group on the health of patients with inflammatory bowel disease and arthritis. Psychol Res Behav Manag.

[36] Berding A, Witte C, Gottschald M, Kaltz B, Weiland R, Gerlich C, Reusch A, Kruis W, Faller H (2017). Beneficial effects of education on emotional distress, self-management, and coping in patients with inflammatory bowel disease: a prospective randomized controlled study. Inflamm Intest Dis.

[37] Zhou Y, Ma X, Chen Y (2014). Dietary practices of Chinese patients with inflammatory bowel disease: a naturalistic inquiry. Gastroenterol Nurs.

[38] Larussa T, Suraci E, Marasco R, Imeneo M, Abenavoli L, Luzza F (2019). Self-prescribed dietary restrictions are common in inflammatory bowel disease patients and are associated with low bone mineralization. Medicina (Kaunas).

[39] Cohen AB, Lee D, Long MD, Kappelman MD, Martin CF, Sandler RS, Lewis JD (2013). Dietary patterns and self-reported associations of diet with symptoms of inflammatory bowel disease. Dig Dis Sci.

[40] Jowett SL, Seal CJ, Pearce MS, Phillips E, Gregory W, Barton JR, Welfare MR (2004). Influence of dietary factors on the clinical course of ulcerative colitis: a prospective cohort study. Gut.

[41] Jantchou P, Morois S, Clavel-Chapelon F, Boutron-Ruault M, Carbonnel F (2010). Animal protein intake and risk of inflammatory bowel disease: The E3N prospective study. Am J Gastroenterol.

[42] Chen J, Wellens J, Kalla R, Fu T, Deng M, Zhang H, Yuan S, Wang X, Theodoratou E, Li X, Satsangi J (2022). Intake of ultra-processed foods is associated with an increased risk of Crohn's disease: a cross-sectional and prospective analysis of 187,154 participants in the UK Biobank. J Crohns Colitis.

[43] Rocha R, Sousa UH, Reis TLM, Santana GO (2019). Nutritional status as a predictor of hospitalization in inflammatory bowel disease: A review. World J Gastrointest Pharmacol Ther.

[44] Lim H, Kim S, Hong S (2018). Food elimination diet and nutritional deficiency in patients with inflammatory bowel disease. Clin Nutr Res.

[45] Brasil Lopes M, Rocha R, Castro Lyra A, Rosa Oliveira V, Gomes Coqueiro F, Silveira Almeida N, Santos Valois S, Oliveira Santana G (2014). Restriction of dairy products; a reality in inflammatory bowel disease patients. Nutr Hosp.

[46] Ostrovsky AM, Chen JR (2020). TikTok and its role in covid-19 information propagation. J Adolesc Health.

[47] Suarez-Lledo V, Alvarez-Galvez J (2021). Prevalence of health misinformation on social media: systematic review. J Med Internet Res.

[48] Armstrong AW, Kim RH, Idriss NZ, Larsen LN, Lio PA (2011). Online video improves clinical outcomes in adults with atopic dermatitis: a randomized controlled trial. J Am Acad Dermatol.

[49] Molavynejad Shahram, Miladinia Mojtaba, Jahangiri Mina (2022). A randomized trial of comparing video telecare education vs. in-person education on dietary regimen compliance in patients with type 2 diabetes mellitus: a support for clinical telehealth providers. BMC Endocr Disord.

[50] Carlsen K, Jakobsen C, Houen G, Kallemose T, Paerregaard A, Riis LB, Munkholm P, Wewer V (2017). Self-managed eHealth disease monitoring in children and adolescents with inflammatory bowel disease: a randomized controlled trial. Inflamm Bowel Dis.

[51] Cross RK, Langenberg P, Regueiro M, Schwartz DA, Tracy JK, Collins JF, Katz J, Ghazi L, Patil SA, Quezada SM, Beaulieu D, Horst SN, Russman K, Riaz M, Jambaulikar G, Sivasailam B, Quinn CC (2019). A randomized controlled trial of TELEmedicine for patients with inflammatory bowel disease (TELE-IBD). Am J Gastroenterol.

[52] Del Hoyo J, Nos P, Faubel R, Muñoz Diana, Domínguez David, Bastida G, Valdivieso B, Correcher M, Aguas M (2018). A web-based telemanagement system for improving disease activity and quality of life in patients with complex inflammatory. J Med Internet Res.

[53] McCombie A, Walmsley R, Barclay M, Ho C, Langlotz T, Regenbrecht H, Gray A, Visesio N, Inns S, Schultz M (2020). A noninferiority randomized clinical trial of the use of the smartphone-based health applications IBDsmart and IBDoc in the care of inflammatory bowel disease patients. Inflamm Bowel Dis.

[54] Heida A, Dijkstra A, Muller Kobold Anneke, Rossen JW, Kindermann A, Kokke F, de Meij Tim, Norbruis O, Weersma RK, Wessels M, Hummel T, Escher J, van Wering Herbert, Hendriks D, Mearin L, Groen H, Verkade HJ, van Rheenen Patrick F (2018). Efficacy of home telemonitoring versus conventional follow-up: a randomized controlled trial among teenagers with inflammatory. J Crohns Colitis.

[55] Akobeng AK, O'Leary N, Vail A, Brown N, Widiatmoko D, Fagbemi A, Thomas AG (2015). Telephone consultation as a substitute for routine out-patient face-to-face consultation for children with inflammatory bowel disease: randomised controlled trial and economic evaluation. EBioMedicine.

[56] Elkjaer M, Shuhaibar M, Burisch J, Bailey Y, Scherfig H, Laugesen B, Avnstrøm Søren, Langholz E, O'Morain C, Lynge E, Munkholm P (2010). E-health empowers patients with ulcerative colitis: a randomised controlled trial of the web-guided 'Constant-care' approach. Gut.

