# Supplementary material for: The Reliability and Quality of Short Videos as a Source of Dietary Guidance for Inflammatory Bowel Disease: Cross-sectional Study
Source: J Med Internet Res. 2023 Feb 9;25:e41518. doi: 10.2196/41518 (PMC9951074; doi:10.2196/41518)
Supplement: Multimedia Appendix 1 [file jmir_v25i1e41518_app1.docx]

**Supplementary Table 1: Modified DISCERN quality criteria for assessing the reliability of video. (1 point for answer ‘yes’, 0 point for answer ‘no’)**

| **Reliability Score** |
| --- |
| 1. Is the video clear, concise, and understandable? |
| 2. Are valid sources cited? |
| 3. Is the content presented balanced and unbiased? |
| 4. Are additional sources of content listed for patient reference? |
| 5. Are areas of uncertainty mentioned? |
