# Supplementary material for: The Reliability and Quality of Short Videos as a Source of Dietary Guidance for Inflammatory Bowel Disease: Cross-sectional Study
Source: J Med Internet Res. 2023 Feb 9;25:e41518. doi: 10.2196/41518 (PMC9951074; doi:10.2196/41518)
Supplement: Multimedia Appendix 2 [file jmir_v25i1e41518_app2.docx]

**Supplementary Table 2: Global Quality Score (GQS) (Scoring ranges from 1 to 5)**

| **GQS Definition** | **Score** |
| --- | --- |
| Poor quality, poor flow of the video, most information missing, not at all useful for patients | 1 |
| Generally poor quality and poor flow, some information listed but many important topics missing, of very limited use to patients | 2 |
| Moderate quality, some important information is adequately discussed | 3 |
| Good quality good flow, most relevant information is covered, useful for patients | 4 |
| Excellent quality and flow, very useful for patients | 5 |
